# Case of Obstinate Costiveness, Terminating Favourably, without the Patient Passing Any Figured Excrement

**Published:** 1818-01

**Authors:** Matthias Rowe


*For the London Medical and Physical Journal.* √

### *Case of obstinate Costiveness, terminating favourably, without the Patient passing any figured Excrement.* By MATTHIAS ROWE, Esq.

THAT Nature is a very useful and powerful auxiliary in the cure of disease cannot be doubted; and whether, in this case, she or the remedies employed were principally instrumental in preserving the life of my patient may be questioned; but I trust the following observations will be considered of sufficient interest to merit insertion in your valuable repository of medical information.

On the 9th of June last, I was sent for to Miss C. about 25 years of age, of spare habit, but generally enjoying pretty good health. I found her labouring under violent pain about the region of the stomach, which was tender when pressed, apparently arising more from spasm than any other cause. With a view of evacuating the bowels, I ordered a draught, with three drachms of sulphat of magnesia, ten grains of jalap, half a dram of compound æther, and one ounce of infusion of senna, to be taken directly; and repeated in two or three hours, if required. The first draught was given at five o'clock in the evening; the second at eight,

I saw her again about ten, when no stool had passed, but the pain was very much abated, though some tenderness remained. I now gave three pills, containing eight grains of calomel, the same quantity of extract of colocynth, two grains of gamboge, and two of antimonial powder; and ordered a draught every three or four hours, composed of two drachms of soda tartarizata and of tincture of jalap, in one ounce of infusion of senna. By eight o'clock on the succeeding morning she had taken three of these draughts, but no discharge from the bowels had followed. The pain was, however, quite gone, and very little soreness remained; the pulse was not more than 75, and soft. I now ordered a fomentation to the abdomen, the use of an enema, and the draught to be continued; of which she had taken three by the evening, when she remained much the same.

On the 11th (the third day), the draughts were again repeated, an enema injected, with half an ounce of spirits of turpentine, and the fomentation continued. During this day two draughts only were taken; the bowels still remained unmoved; pulse 78, and spirits pretty good. At eleven in the evening a draught was given, containing half an ounce of castor oil; to be repeated in the morning, if required. She had some sleep during the night. On the 12th, she was much the same as on the preceding day; the draught, with the castor oil, and the turpentine clyster, was repeated.

I now expressed a wish for further medical advice, and a physician, a friend of the family, met me about five P.M. After hearing the history of my patient, and what had been done for her, he ordered a pill to be given every hour, containing four grains of extract of colocynth and two of calomel, and the use of the simple enemas to be continued; and also prescribed a wine-glass-full of a mixture composed of senna, salts, and manna, to be taken frequently. The warm-bath was had recourse to this evening, until fainting was produced. By the next day, the 13th, twelve of the pills ordered yesterday had been taken. An enema, which was administered the first thing in the morning, came away some little time after, with a small quantity of black fluid, evidently bilious, but not in the least degree fetid. The physician saw her again at two o'clock P. M. and wished two pills to be given, composed of eight grains of calomel and extract of colocynth, and the pill prescribed yesterday to be continued every two hours, with occasionally a draught containing half an ounce of castor oil. By eleven o'clock in the evening, in addition to the two pills given at two in the afternoon, ten of the others had been also taken, and two draughts; yet hitherto not any thing of a satisfactory nature had passed. I now prescribed another turpentine clyster, which came away in about an hour, soon after followed with about half a pint of the same kind of black fluid which had passed in the morning, and equally without odour.

The doctor wishing some small doses of eleterium to be given during the night, if the bowels should not be sufficiently moved, I procured some fresh extract from the Hall, and gave three pills in the course of six hours, each containing half a grain, with three grains of aromatic powder. These produced violent sickness, and were in consequence discontinued. By this time my patient's pulse was beating 110 in the minute, with profuse perspirations, and troublesome hiccough.

After the sickness had abated, she slept for three or four hours, on the morning of the 14th, awoke a good deal relieved, and soon after passed a little more black fluid of the same nature as before. This morning the warm-bath was again used, and an enema administered. No medicine was given during the day, as the stomach rejected every thing of that nature; but a little broth, taken several times, was retained.

For the last twelve hours, perspirations had been very profuse. In the evening she was very ill, complaining of a dead weight and coldness about the abdomen; the pulse 110, and very languid; the hiccough still troublesome. Several times during this evening she passed small quantities of the same black fluid, still devoid of fetor. Considering the danger very much encreased, I felt more and more anxious to relieve the bowels, and injected during the night a weak tobacco clyster, which produced distressing vomiting of matter in appearance like thick black fæces, but having no offensive smell. The pulse was very weak, and beat 130 in the minute. When the sickness abated, profuse perspirations came on, with great coldness and disinclination to move. Indeed, I became a good deal alarmed for her safety, when, to my great satisfaction, she fell asleep, and awoke in about an hour and a half very much better, and able to take a little weak broth. On the morning of the 15th she passed much more black fluid, evidently bile, and still quite free from any smell resembling fæces. Her physician saw her again this morning, and wished her only to take a dose of infusion of roses and sulphat of magnesia every three or four hours, and light nourishing aliment. In the afternoon, offensive matter passed, less fluid than that evacuated before, but quite free from any thing like scybala. From this time (the sixth day of her illness) she began to recover, and the bowels continued to be relieved two or three times a day; but no hard matter ever passed, whereby any satisfactory cause could be assigned for the strange obstruction and other symptoms which had presented themselves. In a few days she was convalescent, and is now in the country enjoying good health.

1, *Clarendon-square, Somers Town;*
*Dec.* 5, 1817.

